# Economic policy uncertainty, renewable energy and environmental degradation: Evidence from Egypt

**DOI:** 10.1007/s11356-023-26426-2

**Published:** 2023-03-29

**Authors:** Mousa Gowfal Selmey, Ahmed A Elamer

**Affiliations:** 1 Brunel Business School, Brunel University London, Kingston Lane, Uxbridge, UB8 3PH London UK; 2 Faculty of Commerce, Mansoura University, Mansoura, Egypt

**Keywords:** Economic policy uncertainty, ARDL, Renewable energy, Sustainable development, Environmental degradation

## Abstract

This study contributes to the growing but still limited body of literature on the impact of economic policy uncertainty, renewable energy usage, and economic growth on environmental degradation in Egypt. Using the autoregressive distributed lag (ARDL) bound test, we examine the existence of cointegration relationships in Egypt over the period 1990–2018. Our results indicate that economic policy uncertainty is positively associated with environmental degradation in both the short and long run. Additionally, we find that economic growth exacerbates environmental degradation in both the short and long term. Finally, renewable energy consumption has a significant negative effect on environmental degradation in the long run. Therefore, ensuring economic policy stability is crucial for maintaining environmental quality. To this end, Egyptian policymakers should prioritize low-carbon research and development techniques, as well as the adoption of renewable energy sources to mitigate environmental degradation.

## Introduction

Over the past few decades, the world has witnessed a series of major challenges that have resulted in political and economic uncertainty worldwide. Examples include the COVID-19 pandemic (Sendstad and Chronopoulos 2020), the Arab Spring revolutions in Middle Eastern countries, and events in Europe such as Russia’s annexation of Crimea, Brexit, the recent Russian invasion of Ukraine, and the refugee crisis. These events have raised concerns about the future of the Euro and European economic policies, in addition to fluctuations in world oil prices (Al-Thaqeb and Algharabali 2019). In the USA, following the recent global financial crisis, policymakers are increasingly concerned about uncertain policies, particularly those related to financial decisions and economic policies (Baker et al. 2016; Wei et al. 2021). Moreover, reports from the International Monetary Fund (IMF) suggest that economic policy uncertainty (EPU) is a crucial factor in depressing the economic growth of a country (Anser et al. 2021).

Given the significance of economic uncertainty, numerous academic studies have explored how EPU affects various economic variables, such as economic growth (Amarasekara et al. 2022; Bhagat et al. 2016; Wen et al. 2022), energy markets (Hailemariam et al. 2019), stock markets (Li et al. 2016; Rehman and Apergis 2019), investment (Kang et al. 2014; Guo et al. 2020; Liu and Zhang 2022), tourism (Akadiri et al. 2020; Liu et al. 2021), and exchange rate volatility (Abid 2020; Krol 2014). In addition to its economic implications, several studies have also suggested that economic uncertainty may have environmental impacts, particularly in light of the global focus on environmental issues as a key aspect of sustainable development. EPU may induce manufacturers to use traditional and environmentally harmful manufacturing processes, leading to environmental degradation (Anser et al. 2021).

EPU may have a variety of effects on environmental degradation. On the one hand, an increase in EPU may lead to a decrease in investment, consumption, and production, resulting in reduced CO_2_ emissions. On the other hand, it may also affect research and development techniques, innovation, and the use of renewable energy, ultimately leading to higher CO_2_ emission levels. As such, EPU can either exacerbate or mitigate the effects of environmental degradation, highlighting the need for research on the relationship between EPU and CO_2_ emissions to inform environmental policies (Anser et al. 2021).

There is a plethora of literature that reveals how the effects of EPU on environmental degradation vary among economies in both the short and long runs. For instance, Adedoyin and Zakari (2020) found that in the short run, EPU reduces environmental degradation, while in the long run, it increases it in the USA. Yu et al. (2021) also observed a positive association between EPU and carbon output in the Chinese manufacturing sector. Shabir et al. (2022) found that EPU has a negative impact on environmental quality in both developing and developed economies.

**Fig. 1:**
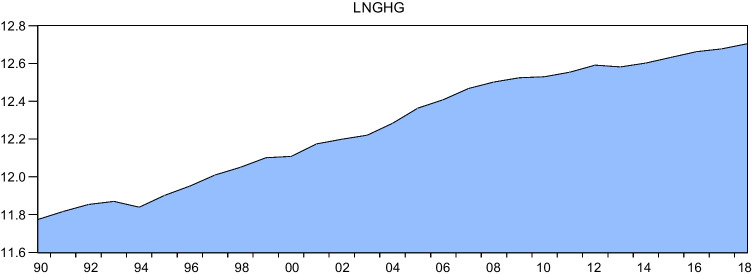
GHG emissions during the period from 1990 to 2018 in Egypt. Sources: World Development Indicators database

**Fig. 2:**
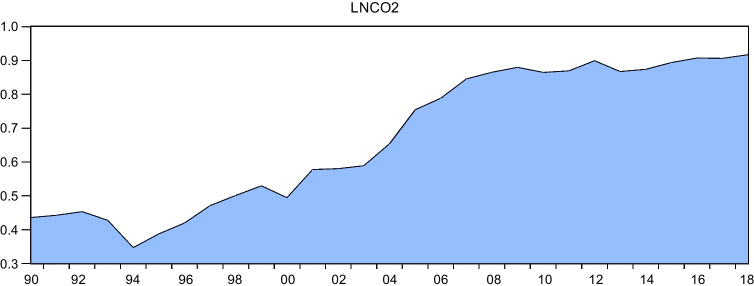
CO_2_ emissions during the period from 1990 to 2018 in Egypt. Sources: World Development Indicators database

**Fig. 3:**
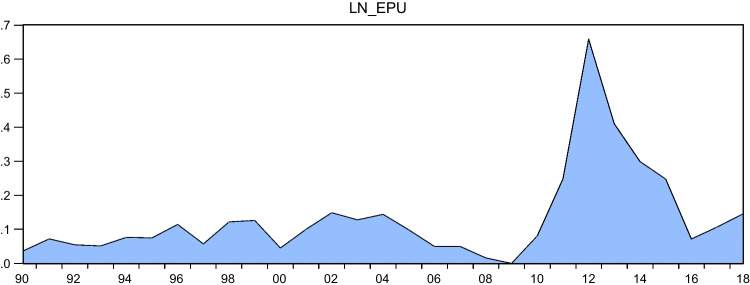
EPU during the period from 1990 to 2018 in Egypt. Source: https://www.policyuncertainty.com/index.html

Despite the growing body of research on the impact of EPU on environmental degradation, there remains a scarcity of literature on this topic, particularly in relation to developing countries such as Egypt. This paper aims to address this gap by examining the effects of EPU and renewable energy on environmental degradation in Egypt. Egypt provides an appropriate context for investigating the environmental consequences of EPU and renewable energy consumption for several reasons. Firstly, as a developing economy, Egypt seeks to achieve economic development while also considering its impact on the environment, as outlined in the 2030 Agenda for Sustainable Development and Egypt Vision 2030. If there is a trade-off between economic growth and environmental sustainability, Egypt will face a difficult decision in determining the path of its development. Secondly, Figs. 1 and 2 demonstrate that there has been a continual increase in CO_2_ and greenhouse gas emissions from 1990 to 2018. Additionally, since the 2011 Arab Spring, Egypt has experienced high levels of volatility in the World Uncertainty Index (WUI), which serves as an indicator of EPU, as illustrated in Fig. 3. Therefore, it is worthwhile to explore the impact of EPU and renewable energy consumption on GHG and CO_2_ emissions in this context. Despite attempts to identify the primary drivers of environmental degradation in developing and developed economies, such as those made by Wen et al. (2022), there is a severe lack of empirical research on the environmental effects of renewable energy and EPU for the economy of Egypt. Also, while there is ample evidence of the positive relationship between renewable energy consumption and environmental quality, the literature lacks consensus on the magnitude and direction of this relationship. Our study contributes to this literature by exploring the impact of renewable energy consumption on CO_2_ emissions in Egypt.

The present study employs the autoregressive distributed lags (ARDL) and bound analyses, as proposed by Pesaran et al. (2001), to investigate the short- and long-run dynamics between EPU, renewable energy demand, and CO_2_ and GHG emissions. The study finds that EPU has a significant and positive relationship with environmental degradation in both the short and long run. In contrast, the consumption of renewable energy is negatively associated with environmental degradation in the long run. Furthermore, the study finds that economic growth is positively related to environmental deterioration in both the short and long run. These novel findings have important implications for policy recommendations.

This paper makes several contributions to the current literature. Firstly, it investigates the impact of economic policy uncertainty and renewable energy consumption on environmental degradation in the Egyptian economy, which, to our knowledge, has not been studied before. The study fills this research gap by examining the effect of these factors on GHG and CO_2_ emissions. Secondly, previous studies have relied on the EPU index developed by Baker et al. (2016) as a measure of economic policy uncertainty, which only considers uncertainty related to economic policies and is limited to a small group of developed economies. To address these limitations, Ahir et al. (2022) developed the World Uncertainty Indicator (WUI), which covers 143 countries and takes into account both economic and political circumstances. This study employs the WUI as an indicator of economic policy uncertainty. Lastly, Egypt has weak environmental regulations, and the reliance of industries on traditional sources of energy exacerbates environmental degradation. Thus, it is essential to understand the effects of EPU and renewable energy on environmental degradation in Egypt. The study’s findings can guide policymakers in formulating effective policies and strategies to achieve the Sustainable Development Goals and protect the environment.

The remaining four sections of this study are organized as follows: the “Review of literature and hypothesis development” section provides a review of the literature. The “Methodology and data” section presents the methodology and data used in this study. The “Empirical findings and discussions” section presents the results and discussion. Finally, the “Conclusion” section concludes the study.

## Review of literature and hypothesis development

### EPU and environmental degradation

EPU has been the subject of increasing concern globally in recent years (Kwabi et al. 2022a, 2022b; Owusu et al. 2022; Salem et al. 2022; Tan et al. 2022). At the macro level, it is well established that EPU has a significant impact on economic activity, including investment, production, employment, R&D investment, and innovation (Ahmed et al. 2020; Amarasekara et al. 2022; Altig et al. 2020; Dibiasi et al. 2018). In addition, a growing body of literature has focused on the relationship between EPU and environmental degradation. However, the results of existing studies on this topic have been inconclusive.

Several studies have examined the relationship between EPU and CO_2_ emissions, but their findings have been inconclusive. For instance, Syed and Bouri (2022) found a positive relationship between EPU and CO_2_ emissions in the short run, indicating that economic policy uncertainty is responsible for environmental degradation in the short term. However, in the long run, there is a negative relationship between EPU and CO_2_ emissions, suggesting that instability reduces CO_2_ emissions and improves the quality of the environment. Similarly, Anser et al. (2021) found that EPU has a significant effect on CO_2_ emissions in the long and short run, with adverse effects in the short term and positive effects in the long term. Yu et al. (2021) also found a positive relationship between provincial EPU in China and the intensity of carbon emissions from industrial companies, with economic policy uncertainty impacting carbon emissions through the proportion of fossil fuels in overall energy consumption and the intensity of energy in the short run.

Shabir et al. (2022)  used the panel vector error correction model and Granger causality methods to analyze the effects of EPU on CO_2_ emissions in 24 developing and developed economies over the period 2001–2019. They found a bidirectional relationship between EPU and CO_2_ emissions, indicating that economic policy uncertainty negatively impacts the environment. Adedoyin and Zakari (2020) used the autoregressive distributed lag technique (ARDL) and bound test to investigate the role of EPU on CO_2_ emissions in the UK during the period 1985–2017. The model found that EPU has a significant impact on CO_2_ emissions, with the most significant effect observed in the short run. In contrast, Wang et al. (2020) found a positive relationship between EPU and CO_2_ emissions, indicating that uncertainty in economic policy does not stimulate investment in R&D, innovations, and renewable energy, thereby leading to an increase in emissions of CO_2_.

Based on the existing literature, we hypothesize that there is a relationship between EPU and environmental degradation, specifically CO_2_ emissions. This relationship can be positive or negative, depending on the level of EPU and its impact on energy consumption, pollution-intensive product consumption, investment in R&D and innovation, and renewable energy. Specifically, we expect that higher levels of EPU will lead to increased emissions of CO2 in the short run, while in the long run, the relationship between EPU and CO_2_ emissions may be negative. Thus, we hypothesize the following:

- **H**_**1**_: There is a linkage between EPU and environmental degradation.

### Economic growth and environmental degradation

The relationship between economic growth and environmental degradation has garnered much attention from researchers due to the adverse impacts of carbon dioxide emissions on climate change and global warming (Albitar et al. 2021, 2022; Alkaraan et al. 2023; Elmarzouky et al. 2021; Giannopoulos et al. 2022; Karim et al. 2021; Moussa et al. 2022; Sharif et al. 2020). Despite the numerous studies that have examined the causal link between economic activity and environmental quality, the empirical evidence remains mixed (Mohamed Abdul Ghani et al.  2017; Radmehr et al. 2021).

A study by Mikayilov et al. (2018) investigated the impact of economic growth on CO_2_ emissions in Azerbaijan. The study found that in the long run, economic growth significantly and positively affects CO_2_ emissions due to the high returns on oil revenues that have led to the revitalization of polluting industries. As a result, energy consumption and subsequent environmental degradation have increased. The income elasticity of CO_2_ emissions was estimated to be between 0.7 and 0.8%. In contrast, Muhammad (2019) examined the relationship between economic growth, energy consumption, and CO_2_ emissions using panel data from 68 economies spanning 2001 to 2017. The results showed that economic growth positively and significantly affects CO_2_ emissions in developed and Middle Eastern and North African (MENA) economies due to increased energy consumption. However, economic growth had a negative impact on CO_2_ emissions in emerging economies, indicating that other factors, such as technological innovation, may be at play. Furthermore, Acheampong (2018) studied the linkage between economic growth, CO_2_ emissions, and energy use across 116 nations between 1990 and 2014. The results suggested that economic growth reduces CO_2_ emissions in the Caribbean and Latin America. However, in other regions, including developed and emerging economies, no significant causal relationship between economic growth and CO_2_ emissions was observed.

Thus, despite the extensive research conducted on the relationship between economic growth and environmental degradation, the empirical evidence remains mixed. Therefore, the present study aims to contribute to the literature by investigating the causal relationship between economic growth and environmental degradation in a specific region or country, providing valuable insights for policymakers to develop effective environmental policies that promote sustainable economic growth. Thus, we hypothesize the following:

- **H**_**2**_: There is a connection between economic growth and environmental degradation.

### Renewable energy consumption and environmental degradation

Renewable energy constitutes a means to preserve the environment, mitigate global warming, and provide energy sources to achieve sustainable development. Hence, in recent decades, there has been a growing interest from researchers and policymakers in the impact of renewable energy consumption on environmental degradation, particularly in the context of global warming and the pursuit of sustainable development (Abdou et al. 2023; Amin et al. 2022; Boulhaga et al. 2023; Elamer et al. 2022; Eldaly et al. 2022; Ibrahim et al. 2022; Murphy et al. 2023; Roberts et al. 2022; Ullah et al. 2022). While various studies have investigated this link, there is still a lack of consensus on the precise relationship between renewable energy use and environmental quality (e.g., Dong et al. 2020).

Koengkan (2018) used the autoregressive distributed lag cointegration method to analyze the link between renewable energy use and environmental degradation in the MERCOSUR countries from 1980 to 2014. The results revealed that renewable energy consumption reduces CO_2_ emissions and thus positively affects the quality of the environment. However, the impact is limited. Similarly, Sharif et al. (2020) investigated the association between renewable energy use and environmental degradation in the top 10 most polluted economies. They found that renewable energy negatively affects environmental degradation in all economies, except for Russia, Indonesia, and India. Nonetheless, there are significant variances across countries, and the positive impact of renewable energy consumption on carbon dioxide may be noticeable in some countries where the costs of implementing renewable energy are offset by utilizing inexpensive fossil fuel energy in other parts of the country. Leitão and Lorente (2020) studied the relationship between renewable energy use and carbon dioxide emissions in the EU. Their findings demonstrated that renewable energy consumption reduces carbon dioxide emissions and mitigates climate change. Finally, Yuping et al. (2021) investigated the role of renewable energy use in Argentina’s carbon dioxide emissions from 1970 to 2018. They found that renewable energy use reduces carbon dioxide emissions in both the short and long run.

Based on these studies, we hypothesize that renewable energy consumption is associated with reduced environmental degradation, particularly in terms of carbon dioxide emissions. However, the impact may be limited in some countries, and the benefits of renewable energy use may vary depending on factors such as economic growth, energy efficiency, and the costs of implementing renewable energy sources. Further research is needed to explore this relationship in greater detail and to develop policies that can help promote the widespread adoption of renewable energy sources to achieve environmental sustainability. Thus, we hypothesize the following:

- **H**_**3**_: There is an association between renewable energy and environmental degradation.

## Methodology and data

### Econometric model

To investigate the effects of economic policy uncertainty (EPU), renewable energy consumption (REC), economic growth (GDP), trade openness (TR), and foreign direct investment (FDI) on Egypt’s greenhouse gas (GHG) emissions, we modeled GHG emissions as a linear function of the explanatory variables in the baseline model, as represented in Eq. (1).

1 $${\mathrm{lndeg}}_{t}={\beta }_{0}{+\beta }_{1}{\mathrm{lnEPU}}_{t}{+\beta }_{2}{\mathrm{lnGDP}}_{t}+{\beta }_{3}{\mathrm{lnREC}}_{t}+{\beta }_{4}{\mathrm{lnFDI}}_{t}+{\beta }_{5}{\mathrm{lnTR}}_{t}+{\varepsilon }_{t}$$

where $\mathrm{deg}$ indicates environmental degradation measured by total emissions of greenhouse gases in kt of CO_2_ equivalent. It is symbolized by GHG. EPU refers to the World Uncertainty Index, which is used as an indicator of uncertainty in economic policy. GDP indicates real economic growth (% annual). REC indicates renewable energy consumption (measured by % of total final energy consumption), FDI refers to foreign direct investment (% of GDP), and TR indicates trade openness (trade % GDP). $\mathrm{ln}$ is a natural log, the parameter ${\beta }_{0}$ refers to the intercept, and ${\beta }_{1}$, ${\beta }_{2}$, ${\beta }_{3}$, ${\beta }_{4}$, and ${\beta }_{5}$ are the elasticities of environmental degradation (measured by total greenhouse gas emissions) that are predicted. The subscript $t$ refers to the period study, while ${\varepsilon }_{t}$ shows the model error term.

### Data collection

This study employs annual time series data from 1990 to 2018 to examine the impacts of economic policy uncertainty (EPU), renewable energy consumption (REC), economic growth (GDP), trade openness (TR), and foreign direct investment (FDI) on Egypt’s greenhouse gas (GHG) emissions. The data is sourced from the World Bank’s World Development Indicator, with additional data on economic policy uncertainty collected from the World Uncertainty Index. GHG emissions are treated as the dependent variable, while the aforementioned variables serve as explanatory variables in the model.

### Econometric methodology

In this study, we employ an augmented autoregressive distributed lag (ARDL) and bounds test approach to investigate the impact of economic policy uncertainty (EPU) and renewable energy consumption (REC) on environmental degradation in Egypt. This model, originally proposed by Pesaran and Shin (1999) and Pesaran et al. (2001), has gained popularity due to its ability to overcome the limitations of traditional cointegration tests. These tests, such as the Engle-Granger test (1987) and the Johansen test (1991, 1995), require that the variables under consideration be non-stationary and integrated in the same order. In contrast, the ARDL model can accommodate stationary and non-stationary time series with different integration orders, as long as the dependent variable is integrated of order one (I(1)) (Pesaran et al. 2001).

Furthermore, this cointegration technique enables us to examine both the long- and short-run relationships among non-stationary series and to model them using the error correction model (Nkoro and Uko 2016). We will apply this methodology to our annual time series data, spanning from 1990 to 2018, for GHG emissions, EPU, REC, economic growth (GDP), trade openness (TR), and foreign direct investment (FDI), which were obtained from the World Development Indicator of the World Bank. Additionally, data on economic policy uncertainty was collected from the World Uncertainty Index. By analyzing the relationships between these variables, we aim to provide insights into the potential effects of economic policy uncertainty and renewable energy use on environmental degradation in Egypt.

The next step in the analysis involves estimating the model using several techniques. First, the model is estimated using the ARDL and bound test approach, which was proposed by Pesaran and Shin (1999) and Pesaran et al. (2001). This method is preferred because it can be used for both stationary and non-stationary time series with various integration orders, and it allows for the inclusion of I(0) and I(1) variables. In addition, the dependent variable must be integrated in order 1, or I(1). The bound test approach is used to determine the existence of cointegration between the variables, which is a necessary condition for estimating long-run relationships.

Second, the error correction model (ECM) is used to estimate the short-run dynamics of the model. The ECM captures the adjustment process that occurs when the dependent variable deviates from its long-run equilibrium. The ECM is important in this study because it allows us to determine how quickly the system will converge back to its long-run equilibrium after a shock.

Finally, several diagnostic tests are conducted to ensure the validity of the model. These tests include the tests for serial correlation, normality, heteroscedasticity, and stability of the model coefficients. The results of these tests help to determine the suitability of the model for making inferences and drawing conclusions.

#### ARDL and bound test

Cointegration testing using a model of the general ARDL (*p*, *q*1, *q*2, ......, *qk*):

3 $$\Delta {Y}_{t}={\delta }_{0t}+\sum_{i=1}^{k} {\alpha }_{i}\Delta {Y}_{t-1}+\sum_{i=1}^{k} {\alpha }_{2}\Delta {X}_{t-i}+{\delta }_{l}{Y}_{t-1}+{\delta }_{2}{X}_{t-1}+{v}_{lt}$$

where *k* is the ARDL model’s maximum lag order, *Δ* indicates the first difference, *Y* represents the dependent variable, and *X* is an exogenous explanatory variable. The F-statistic is utilized to test the joint hypotheses (h0) that the coefficients of the lagged variables ${(\delta }_{1}{\mathrm{X}}_{t-1}{\delta }_{1}{\mathrm{Y}}_{t-1}\text{ or }{\delta }_{1}{\mathrm{Y}}_{t-1}{\delta }_{1}{\mathrm{X}}_{t-1})$ are zero. (*δ*1 – *δ*2) denotes the long-run relation, whereas (*α*1–*α*2) denotes the short-run dynamics of the model (Nkoro and Uko 2016).

The ARDL bound test indicates that whenever the calculated F-statistic is greater than the upper bound critical value, the null hypothesis (H0) of no cointegration is rejected. If the calculated F-statistic is less than the critical value for the lower bound, the H0 cannot be rejected, indicating that the variables are not cointegrated. However, the bounds test’s calculated F-statistics are invalid if the variables are stationary at a level higher than I(1). Therefore, the F-statistics values presented by Pesaran et al. (2001) cannot be applied in such cases. In the next step, we estimate the long-run relationship of the variables using the long-run equation for the ARDL model, which takes the following form:

4 $${Y}_{t}={\delta }_{0}+\sum_{i=1}^{k} {\alpha }_{1}{X}_{1t}+\sum_{i=1}^{k} {\alpha }_{2}{X}_{2t}+\sum_{i=1}^{k} {\alpha }_{3}{X}_{3t}+\sum_{i=1}^{k} {\alpha }_{n}{X}_{nt}+{v}_{1t}$$

*k* is the number of optimum lag orders. $X\mathrm{s }\left({X}_{1t},{X}_{2t},{X}_{3t},\dots \dots \dots .,{X}_{\mathrm{n}t}\right)$ are the explanatory or long-run forcing variables. The better model estimates the associated error correction model (ECM).

#### The error correction model (ECM)

In the circumstance of cointegration, we use the autoregressive distributed lag (ARDL) error correction model to scrutinize long-run relationships and short-run dynamics. The error correction model (ECM) equation is as follows:

5 $$\Delta {Y}_{t}=\mu +\sum_{i=1}^{n-1} {a}_{i}\Delta {Y}_{t-i}+\sum_{i=0}^{m-1} {\gamma }_{i}\Delta {X}_{t-i}-\lambda E{C}_{t-l}+{\varepsilon }_{t}$$

The most important interpretation of this equation is *λ*, which refers to the error correction coefficient or adjustment coefficient. In addition, the error correction term should be statistically significant and negative, as it indicates how quickly variables reach equilibrium in the long run (Okoro et al. 2021). This is commonly referred to as the “speed of adjustment.” The error correction term also shows the percentage of disequilibrium that is being corrected, which represents how much of the prior period’s disequilibrium is being rectified or how much adjustment to equilibrium is taking place in each period. If the estimate of $\lambda {\mathrm{EC}}_{t-l}$ =1, it means that 100% of the adjustment occurs during the period of ${Y}_{t}$. If the estimate of $\lambda {\mathrm{EC}}_{t-l}$ =0.5, it shows that 50% of the adjustment occurs during the period of ${Y}_{t}$. If the estimate of $\lambda E{C}_{t-l}$ =0, it indicates that there is no adjustment, and it is no longer logical to have a long-run relationship (Nkoro and Uko 2016).

## Empirical findings and discussions

### Main findings

#### Stationary check

The results of the augmented Dickey-Fuller test are presented in Table 1. The test indicates that all variables are in level-stationary form except for lnGHG and lnEPU, which are stationary at the first difference. The test also shows that lnGDP is stationary at a 1% level, while lnFDI and lnGDP are stationary at a 5% level. Similarly, lnREC and lnTR are stationary at a 10% level. The first difference of lnGHG and lnEPU are stationary at the 1% level, making them integrated at order one (I(1)). The results of the Phillips-Perron test corroborate those of the augmented Dickey-Fuller test, except for lnTR, which is stationary at the 5% level at the first difference. Moreover, the residual series is stationary at the 1% level. All data series have a mixed order of (1) and level, and the residual series is stationary in level. Therefore, the ARDL bound cointegration test can be used.

**Table 1:** Stationary test

Variables	Augmented Dickey-Fuller test			Phillips-Perron test		
	At I(0)	At I(1)	Decision	At I(0)	At I(1)	Decision
lnGHG	− 0.856	− 4.475^**^*	Stationary at I(1)	− 1.00	− 4.475^***^	Stationary at **I(1)**
lnEPU	− 2.510	− 4.782^**^*	Stationary at I(1)	− 2.691	− 4.769^***^	Stationary at **I(1)**
lnGDP	3.708^***^		Stationary at I(0)	− 3.733^***^		Stationary at **I(0)**
lnREC	− 3.5^*^		Stationary at I(0)	− 3.486^*^		Stationary at **I(0)**
lnFDI	− 2.234^**^		Stationary at I(0)	− 2.234^**^		Stationary at **I(0)**
lnTR	− 3.614^*^		Stationary at I(0)	− 1.898	− 4.298^**^	Stationary at **I(1)**
Res(Ui)	− 6.903^***^		Stationary at I(0)			

*, **, and *** indicate significance at 10%, 5%, and 1%, respectively

#### The ARDL cointegration method

The first step of the ARDL bound cointegration test is to determine the optimal lag length that minimizes the information criteria, including the Schwarz information criterion (SC), Hannan-Quinn information criterion (HQ), and Akaike information criterion (AIC) of the unrestricted VAR approach. As shown in Table 2, the optimal lag length is found to be one. Subsequently, Table 3 reveals that the calculated F-statistic (5.837) for the bound test exceeds the upper bound critical value at the 1% level (5.419), 5% level (4.013), and 10% level (3.417). These results indicate that the variables have a cointegration relationship in the long run. Therefore, the ARDL-ECM technique can be used for both short-run and long-run analyses.

**Table 2:** Optimal lag length

Lag	LogL	AIC	SC	HQ
0	34.188	− 2.088	− 1.8	− 2.002
1	145.883	− 7.695^*^	− 5.679^*^	− 7.096^*^
2	178.813	− 7.468	− 3.724	− 6.354

* indicates the lag order that the criterion selected

**Table 3:** Test of bound cointegration—ARDL model

Test	Value	Sig	I (0)	I(1)
F-statistic	5.837	10%	2.331	3.417
		5%	2.804	4.013
		1%	3.9	5.419

#### ARDL long run and ECM findings

Table 4 displays the short-run and long-run results. The empirical findings suggest that economic policy uncertainty (EPU) has a significant positive relationship with environmental degradation in both the short run and long run. Specifically, a 1% increase in EPU leads to a 0.374% increase in total greenhouse gas emissions in the long run at a 5% significance level. This finding aligns with the previous studies, including Aliyu et al. (2018), who observed that the unstable economic policies adopted by the Egyptian government during the study period, combined with multiple crises such as the global financial crisis in 2008 and the Arab Spring revolutions in 2011, resulted in less attention given to environmental measures. As a result, individuals and companies relied more on cheap traditional energy sources such as coal and oil, which are major contributors to greenhouse gas emissions. Furthermore, high EPU can negatively impact renewable energy investments in Egypt, leading to further reliance on fossil fuels (Ayad et al. 2023).

**Table 4:** ARDL long run/ECM findings

Variable	Coefficient	T-statistic	Prob
Long run			
*lnEPU*	0.374	2.161	0.046^**^
*lnGDP*	0.313	3.31	0.0044^***^
*lnREC*	− 1.319	− 13.980	0.0000^***^
*lnFDI*	− 0.042	− 1.966	0.0669^*^
*lnTR*	0.186	1.793	0.092^*^
*C*	13.724	33.152	0.0000^***^
ECM and short run			
*D(lnGHG(-1))*	− 0.329	− 2.051	0.057^*^
*D(ln_EPU)*	0.058	1.982	0.065^*^
*D(lnGDP)*	0.059	5.188	0.0001^***^
*D(ln_FDI)*	− 0.004	− 1.587	0.132
*ECT (− 1)*	− 0.334	− 7.495	0.0000^***^

*, **, and *** indicate significance at 10%, 5%, and 1%, respectively

This result is also consistent with other studies, such as Anser et al. (2021) and Wang et al. (2020), who found a positive and significant relationship between economic policy uncertainty and environmental degradation. Economic policy uncertainty discourages investment in R&D and innovation, leading to an increase in environmental degradation. In the short run, a 1% increase in EPU results in a 0.058% increase in total greenhouse gas emissions at a 10% significance level. Nakhli et al. (2022) noted that economic policy uncertainty can impact pollution levels by increasing energy use. Specifically, the uncertainty surrounding economic policy can affect the business climate and the decisions made by industries and manufacturing enterprises, which can lead to more carbon emissions. Thus, the results of the study indicate that economic policy uncertainty has a significant positive relationship with environmental degradation in both the short run and long run. The unstable economic policies and crises faced by Egypt during the study period may have contributed to the positive relationship observed between EPU and environmental degradation. The findings of this study highlight the need for stable economic policies and investments in renewable energy sources to reduce environmental degradation.

Similarly, empirical findings indicate a positive and significant association between environmental degradation and economic growth (GDP) in both the short and long run. Specifically, a 1% increase in economic growth results in a 0.313% increase in greenhouse gas (GHG) emissions in the long run and a 0.059% increase in the short run at a significant level of 1%. This implies that economic growth contributes to environmental degradation in Egypt, primarily due to the high dependence on primary energy consumption in the productive sectors of the economy, which negatively impacts environmental quality. This outcome is consistent with earlier studies (Chien et al. 2022; Khan and Ozturk 2020; Leitão and Lorente 2020; Hao et al. 2019) that have found a positive relationship between economic growth and ecological degradation, largely driven by traditional energy consumption in the production of goods and services. In line with this, Yusuf et al. (2020) found that economic growth significantly and positively affects carbon dioxide emissions.

As expected, the use of renewable energy is negatively associated with GHG emissions, indicating that a reduction in renewable energy usage increases environmental degradation in Egypt. Specifically, a 1% reduction in renewable energy use leads to a 1.319% increase in environmental degradation in the long run at a significant level of 1%. This underscores the importance of using and investing in renewable energy to improve environmental quality in Egypt. However, renewable energy investments in Egypt are heavily dependent on government funding, and private sector participation is declining, posing a significant burden on the government budget, especially given the higher investment costs of renewable energy production relative to fossil fuels. This result is consistent with earlier research (Apergis et al. 2023; Adebayo et al. 2022), which found that the use of renewable energy has an adverse impact on carbon dioxide emissions in other countries. Nevertheless, using various renewable energy sources, such as photovoltaic solar energy, wind energy, hydroelectricity, and biofuels, can significantly reduce GHG emissions and lead to energy-saving techniques by reducing non-renewable energy use (Lima et al. 2020).

Moreover, foreign direct investment has a statistically significant negative influence on environmental degradation in the long run at a 10% level in Egypt, while its effect is insignificant in the short run. The result suggests that a 1% change in foreign direct investment decreases GHG emissions by 0.042% in the long run. Finally, the findings reveal that trade openness has a significant positive effect on environmental degradation in the long run at a 10% level in Egypt, consistent with the findings of Mahmood et al. (2019).

The speed of adjustment is determined by the error correction term (ECT). The ECM results demonstrate that, as anticipated, the ECT is highly significant and negative at the 1% level. This finding indicates that there exists a stable long-run relationship among lnEPU, lnGDP, lnREC, lnFDI, lnTR, and lnGHG. The ECT value indicates that the rate of adjustment takes approximately 33.4% of a year to achieve equilibrium in the long run. Put another way, the complete adjustment occurs over approximately the next 3 years to achieve long-run equilibrium. Another way to interpret this result is that a deviation from the long-run equilibrium level of GHG emissions in a given year is corrected by approximately 33.4% in the subsequent year (Brini et al. 2017). The short-run result shows that past GHG emissions have a positive influence on current emissions, resulting in a yearly decrease of 0.329% in GHG emissions in Egypt (Adedoyin and Zakari 2020).

#### Diagnostic tests

**Fig. 4:**
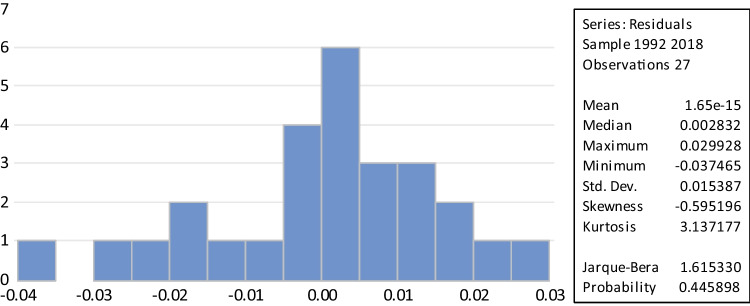
Normality of the residuals

Table 5 presents the results of several diagnostic tests performed on the model. Firstly, the LM test for Breusch-Godfrey serial correlation indicates that the serial correlation does not have autocorrelation. Secondly, the Breusch-Pagan Godfrey test shows the presence of homoskedasticity, indicating no heteroskedasticity in the model. Thirdly, the Jarque–Bera test, with a *p*-value above 5% and Fig. 4, confirms that the data meets the normal distribution requirement.

**Table 5:** Diagnostic tests

Specification	*p*-values	Conclusion
LM Breusch-Godfrey serial correlation test (autocorrelation)	0.282	There is no autocorrelation
Breusch-Pagan-Godfrey heteroskedasticity test		
Jarque–Bera (normality)	0.929	No heteroskedasticity
CUSUM (stability)	0.446	Normality of the residuals
CUSUM of squares (stability)		Stability model
Test of Ramsey RESET		Stability model
	0.194	Model specified correctly

**Fig. 5:**
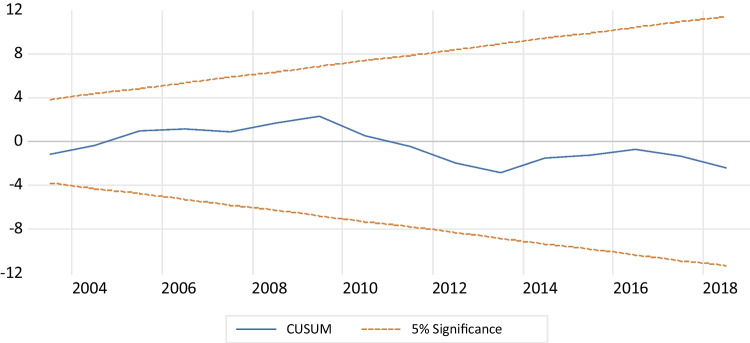
CUSUM for the model. Stability at 5% significance

**Fig. 6:**
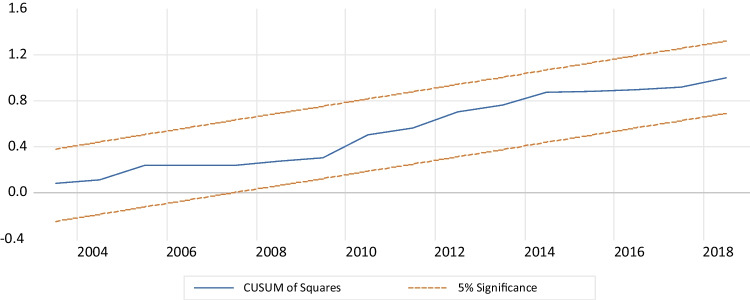
CUSUMSQ for the model. Stability at 5% significance

In addition, the cumulative sum of recursive residuals (CUSUM) and the cumulative sum of squared residuals (CUSUMQ) shown in Figs. 5 and 6, respectively, demonstrate the structural stability of the model. Both CUSUM and CUSUMSQ fall within the critical interval line at a 5% level, indicating that the coefficients were stable throughout the study period and that the model is reliable and accurate (Brini et al. 2017). Furthermore, we employed the Ramsey reset approach to test the accuracy of the autoregressive distributed lag model presentation, and the results confirmed that the estimated ARDL technique was correctly aligned with the data. Overall, the model diagnostics indicate that the ARDL model is suitable for analyzing the relationship between environmental degradation and economic growth in Egypt.

### Additional analyses

In our study, we aimed to investigate the impact of economic policy uncertainty (EPU), renewable energy consumption (REC), foreign direct investment (FDI), economic growth (GDP), and trade openness (TR) on CO_2_ emissions, which are considered as an indicator of environmental degradation, in Egypt. The baseline model assumes that CO_2_ emissions can be expressed as a linear function of the explanatory variables, as shown in the equation below:

$${\mathrm{lndeg}}_{t}={\beta }_{0}{+\beta }_{1}{\mathrm{lnEPU}}_{t}{+\beta }_{2}{\mathrm{lnGDP}}_{t}+{\beta }_{3}{\mathrm{lnREC}}_{t}+{\beta }_{4}{\mathrm{lnFDI}}_{t}+{\beta }_{5}{\mathrm{lnTR}}_{t}+{\varepsilon }_{t}$$

Then, the model is estimated as follows: (a) stationary test, (b) ARDL and bound test, (c) error correction model, and (d) diagnostic test.

#### Stationary check

Table 6 presents the results of the augmented Dickey-Fuller (ADF) test, which shows that all variables, except for lnCO_2_ and lnEPU in level form, are stationary. However, their first differences are stationary, indicating that they are integrated of order one, I(1). The Phillips-Perron (PP) test produced similar results, except for lnTR, which is stationary at the 5% level in its first difference, I(1). Furthermore, the residual series is stationary at the 5% level. All data series are in mixed order (1) and level, and the residual series is stationary in level. These results indicate that the ARDL bound cointegration test can be used.

**Table 6:** Stationary test

Variables	Augmented Dickey-Fuller test			Phillips-Perron test		
	At I(0)	At I(1)	Decision	At I(0)	At I(1)	Decision
lnCO_2_	− 1.654	− 4.289^**^	Stationary at I(1)	− 1.857	− 4.289^**^	Stationary at I(1)
lnEPU	− 2.51	− 4.782^***^	Stationary at I(1)	− 2.691	− 4.769^***^	Stationary at I(1)
lnGDP	3.708^***^		Stationary at I(0)	− 3.733^***^		Stationary at I(0)
lnREC	− 3.501^*^		Stationary at I(0)	− 3.486^*^		Stationary at I(0)
lnFDI	− 2.234^**^		Stationary at I(0)	− 2.234^**^		Stationary at I(0)
lnTR	− 3.614^*^		Stationary at I(0)	− 1.898	− 4.298^**^	Stationary at I(1)
Res(Ui)	− 4.263**		Stationary at I(0)			

*, **, and *** indicate significance at 10%, 5%, and 1%, respectively

#### ARDL cointegration tests

Table 7 shows that the calculated F-statistic value of 5.954 is higher than the upper bound critical values of 5.419 at 1%, 4.013 at 5%, and 3.417 at 10%. This result indicates the existence of a cointegrating long-run relationship among the variables. Therefore, we can employ the ARDL-ECM technique to perform long and short-run analyses.

**Table 7:** Bound cointegration test—ARDL model

Test statistic	Value	Sig	I(0)	I(1)
F-statistic	5.954	10%	2.331	3.417
		5%	2.804	4.013
		1%	3.9	5.419

#### ARDL long run and ECM findings

Table 8 presents the short- and long-term results of the study. The findings suggest that economic policy uncertainty (EPU) has a significant positive effect on CO_2_ emissions in the long run. Specifically, a 1% increase in EPU results in a 0.228% increase in CO_2_ emissions in the long run at a 10% level. Similarly, economic growth (GDP) has a significant positive effect on CO_2_ emissions in both the long and short run. A 1% increase in GDP leads to a 0.085% increase in CO_2_ emissions in the long run at a 10% level, and a 0.023% increase in CO_2_ emissions in the short run at a 5% level. These findings suggest that economic growth has a detrimental effect on the environment. As expected, the results indicate that renewable energy consumption has a negative effect on CO_2_ emissions, meaning that increasing the use of renewable energy reduces CO_2_ emissions in Egypt. A 1% increase in renewable energy use leads to a 0.839% decrease in CO_2_ emissions in the long run at a 1% level. On the other hand, foreign direct investment has an insignificant effect on CO_2_ emissions.

**Table 8:** ARDL long run/ECM results

Variable	Coefficient	T-statistic	Prob
Long run			
*lnEPU*	0.228	2.059	0.053^*^
*lnGDP*	0.085	1.88	0.075^*^
*lnREC*	− 0.839	− 15.57	0.0000^***^
*lnFDI*	0.0003	0.037	0.971
*lnTR*	0.141	2.312	0.032^**^
*C*	1.586	6.127	0.0000^***^
ECM and short run			
*D(lnGDP)*	0.023	2.453	0.024^**^
*ECT (− 1)*	− 0.577	− 7.361	0.0000^***^

*, **, and *** indicate significance at 10%, 5%, and 1%, respectively

Furthermore, the results reveal that trade openness has a significant positive effect on CO_2_ emissions in the long run. A 1% increase in trade openness leads to a 0.141% decrease in CO_2_ emissions in the long run at a 5% level in Egypt. The ECM results suggest that there is a stable long-run relationship between lnEPU, lnGDP, lnREC, lnFDI, lnTR, and lnCO_2_ emissions, with the error correction term (ECT) being highly significant and negative at a 1% level of significance. This indicates that deviations from the equilibrium level of CO_2_ emissions in 1 year are adjusted by 57.7% in the following year. Overall, these results suggest that policies aimed at reducing economic policy uncertainty, promoting the use of renewable energy, and reducing trade openness could help reduce CO_2_ emissions in Egypt.

#### Diagnostic tests

**Fig. 7:**
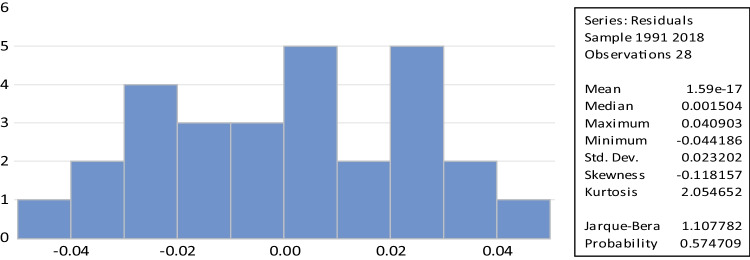
Normality of the residuals

**Fig. 8:**
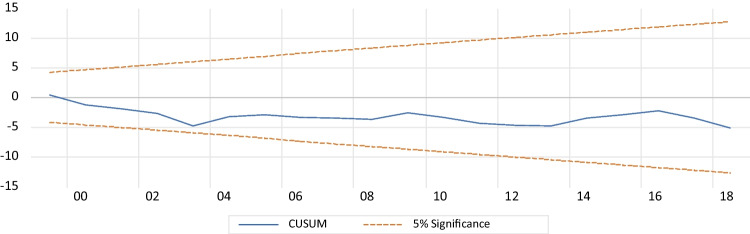
CUSUM for the model. Stability at 5% significance

**Fig. 9:**
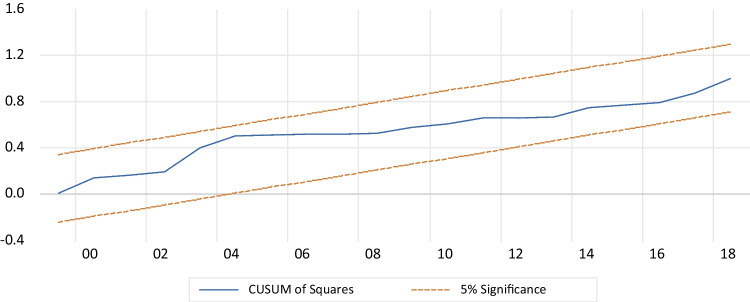
CUSUMSQ for the model. Stability at 5% significance

Table 9 presents the results of various diagnostic tests conducted on the model. The LM test for Breusch-Godfrey serial correlation indicates that there is no serial correlation or autocorrelation. The Breusch-Pagan Godfrey test confirms the presence of homoskedasticity, indicating that there is no heteroskedasticity. The Jarque–Bera test shows that the data meets the normal distribution assumption, as evidenced by the *p*-value being greater than 5% (Fig. 7). Additionally, the cumulative sum of recursive residuals (CUSUM) and the cumulative sum of squared residuals (CUSUMQ) in Figs. 8 and 9 demonstrate the model’s structural stability. Specifically, both CUSUM and CUSUMSQ fall within the critical interval at a 5% level of significance, indicating that the model is stable. Lastly, the Ramsey reset test is used to verify the accuracy of the ARDL model presentation, and the findings confirm that the estimated ARDL model is correctly specified.

**Table 9:** Diagnostic tests

Specification	*p*-values	Conclusion
LM Breusch-Godfrey serial correlation test (autocorrelation)	0.771	There is no autocorrelation
Breusch-Pagan-Godfrey heteroskedasticity test		
Jarque–Bera (normality)	0.589	No heteroskedasticity
CUSUM (stability)	0.575	Normality of the residuals
CUSUM of squares (stability)		Stability model
Test of Ramsey RESET		Stability model
	0.194	Model specified correctly

The recent COVID-19 crisis has brought about unprecedented economic policy uncertainty, which can have significant implications for environmental degradation. According to a study by Acheampong et al. (2020), the COVID-19 pandemic has resulted in a reduction in global carbon emissions due to the slowdown in economic activity and travel restrictions. However, this reduction may be short-lived, as countries focus on economic recovery and may prioritize economic growth over environmental concerns, leading to increased pollution and environmental degradation. Furthermore, the uncertainty surrounding the duration and severity of the pandemic and the effectiveness of policy responses can lead to delays in implementing long-term environmental policies, as governments may focus on short-term economic stabilization measures (Elamer et al., 2022).

In summary, while the COVID-19 crisis may have initially led to a reduction in environmental degradation due to reduced economic activity, the long-term impact of economic policy uncertainty on the environment remains uncertain and dependent on the actions taken by governments and businesses in the post-pandemic recovery period. Therefore, it may not be appropriate to include data from the COVID-19 period in the analysis, as it could bias the results and limit the generalizability of the findings to other time periods. Additionally, the COVID-19 pandemic represents a unique shock to the global economy that is unlikely to be repeated in the future, making it less relevant for modeling long-term trends in environmental degradation.

## Conclusion

In recent decades, economic policy uncertainty (EPU) and renewable energy consumption have experienced a profound upsurge. In addition to the economic impacts, EPU and renewable energy consumption have environmental impacts as well. Based on this, the current paper investigated the effects of EPU (a proxy for the index of world uncertainty) and renewable energy on environmental degradation (measured by GHG emissions) in Egypt over the period from 1990 to 2018. To this end, the study used the dynamic ARDL bound test methodology for cointegration and error correction model (ECM) representation. The main results from our estimation are that first, economic policy uncertainty increases environmental degradation in both the short run and long run. Second, economic growth leads to environmental degradation in both the short run and long run. Lastly, the consumption of renewable energy has a significant adverse effect on environmental degradation in the long run.

Our findings have some important policy repercussions on the energy and carbon market. If Egypt desires to reduce both environmental pollution and EPU at the same time, economic policies must be extremely clear and transparent. Additionally, it should promote innovation, renewable energy, and the use of alternative, job-creating technologies. Specifically, the Egyptian authorities ought to coordinate its economic strategies, particularly the renewable energy policy to attenuate environmental degradation. Our results suggest significant changes in renewable energy policy to deal with economic policy uncertainty. Governments are being urged to provide tax deductions for clean energy use while spending on research and development should be increased. Furthermore, projects and grants for new technologies and clean energy techniques should be given, and subsidies for the import of renewable energy sources should be provided. International organizations also such as United Nations should minimize geopolitical tensions to reduce environmental degradation. Managers of renewable energy companies operating in Egypt should take note of our finding that renewable energy consumption has a significant adverse effect on environmental degradation in the long run. They should consider adopting cleaner and more sustainable energy technologies and reducing the use of fossil fuels to mitigate environmental degradation.

Our study has some limitations that future research could address. First, we used GHG emissions as a measure of environmental degradation, but there are other measures that could be used. Future studies could investigate the impacts of economic policy uncertainty and renewable energy consumption on other measures of environmental degradation such as air pollution or water pollution. Second, our study focused on Egypt, and our results may not be generalizable to other countries. Future studies could investigate the impacts of economic policy uncertainty and renewable energy consumption on environmental degradation in other countries or regions. Third, future studies could investigate the impacts of other variables, such as institutional quality or trade openness, on the relationship between economic policy uncertainty, renewable energy consumption, and environmental degradation.

## Data Availability

Data is available upon request.
